# Investigating Monophyly of Typhlocybini Based on Complete Mitochondrial Genomes with Characterization and Comparative Analysis of 19 Species (Hemiptera: Cicadellidae: Typhlocybinae)

**DOI:** 10.3390/insects14110842

**Published:** 2023-10-30

**Authors:** Xian Zhou, Yuejie Lei, Christopher H. Dietrich, Min Huang

**Affiliations:** 1 Key Laboratory of Plant Protection Resources and Pest Management of Ministry of Education, Entomological Museum, College of Plant Protection, Northwest A&F University, Xianyang 712100, China; zhouxian191@163.com (X.Z.); m18899531077@163.com (Y.L.); 2 Illinois Natural History Survey, Prairie Research Institute, University of Illinois, 1816 S. Oak St., Champaign, IL 61820, USA; chdietri@illinois.edu

**Keywords:** mitochondrial genome, Typhlocybini, phylogenetic analysis

## Abstract

**Simple Summary:**

Typhlocybinae is the second-largest subfamily in the leafhopper family Cicadellidae, comprising approximately 6000 valid species worldwide. Typholocybinae has been divided into six tribes. Typhlocybini and Zyginellini were distinguished only based on one character of the hind wing venation, and the latter tribe was recently considered to be a junior synonym of the former, but few mitogenome sequences representative of major lineages of Typhlocybini have been available to facilitate a comprehensive phylogenetic analysis of the tribe. Thus, we performed complete mitogenome sequencing and functional annotations for 19 species of Typhlocybinae, combining them with 48 mitogenome sequences of previously available typhlocybine mitogenomes to reconstruct phylogenetic relationships with the main lineages to examine the monophyly of Typhlocybini and the phylogenetic status and relationships among generic complexes. The results confirm that Zyginellini should be treated as a junior synonym of Typhlocybini, and some generic groups previously recognized based on morphological characters correspond to monophyletic lineages.

**Abstract:**

Tribes of the leafhopper subfamily Typhlocybinae have traditionally been defined based on differences in forewing and hindwing venation. Except for Typhlocybini (sensu lato), the classification of tribes is relatively stable. The monophyly of Typhlocybini needs to be examined, and the relationships among genera within Typhlocybini have not been resolved. Few mitogenome sequences representative of major lineages of Typhlocybini have been available to facilitate a comprehensive phylogenetic analysis of the tribe. In this study, the complete mitogenomes of 19 species of Typhlocybini were sequenced. The gene arrangements of the 19 new mitogenomes are consistent with ancestral insect mitogenomes. Phylogenetic analyses by both maximum-likelihood and Bayesian methods of 67 species of Typhlocybinae suggest that Zyginellini is paraphyletic with respect to Typhlocybini. The phylogenetic relationships within Typhlocybini are discussed, and the major results show that the *Farynala* and *Linnavuoriana* complexes previously recognized based on morphological characters correspond to monophyletic lineages.

## 1. Introduction

Typhlocybinae is the second-largest membracoid subfamily in the leafhopper family, Cicadellidae, comprising about 6000 valid species worldwide [1,2,3]. These insects are phytophagous and familiar as serious pests in agricultural ecosystems [4,5,6,7,8]. Six tribes (Alebrini, Dikraneurini, Empoascini, Erythroneurini, Typhlocybini and Zyginellini) were recognized in the widely adopted classification of Typhlocybinae [9]. Typhlocybini and Zyginellini were distinguished only based on one character of the hindwing venation: whether or not the submarginal vein is directly connected to vein CuA [10]. Recently, the latter tribe was considered to be a junior synonym of the former [3,11,12].

Phylogenetic studies have generally supported the recognition of five typhlocybine tribes. In the first comprehensive phylogenetic study of the group, Balme (2007) first introduced molecular data into the phylogenetic analysis of Typhlocybinae, he used two molecular markers (*16S* rDNA, *H3*) and discrete morphological characters to obtain a well-resolved estimate, with Dikraneurini paraphyletic with respect to Erythroneurini [2]. Lin et al. (2021) [11] conducted a preliminary investigation on the phylogenetic status of Zyginellini using mitogenomes and recovered Dikraneurini and Erythroneurini as distinct lineages. The results of those analyses also suggested that Zyginellini is derived from within Typhlocybini. Lu et al. (2021) used five genes (*16S* rDNA, *COI*, *H2A*, *H3*, *28S* rDNA D2) to further explore the status and relationships of the tribes in Typhlocybinae and recovered Zyginellini as sister to Typhlocybini, but this result had low branch support, and the dataset included only two genera of Zyginellini [13]. Most recently, Cao et al. (2023) used a large dataset comprising 665 genetic loci and 234 species to obtain a well-resolved phylogeny of the subfamily, consistent with the previous five-tribe system with an additional new tribe, Beameranini, established for a small group of morphologically aberrant Neotropical genera [12]. This analysis showed that Zyginellini is polyphyletic, with different groups of zyginelline genera derived independently within Typhlocybini [12].

Previous analyses have not consistently resolved relationships among genera within Typhlocybini. Thus, the phylogenetic status of some generic groups recognized by previous authors within Typhlocybini needs to be verified. In a morphology-based phylogenetic analysis, Huang (2003) recognized four generic complexes within Typhlocybini sensu stricto, namely the *Eupteryx* complex, *Typhlocyba* complex, *Farynala* complex and *Linnavouriana* complex, with all except the *Typhlocyba* complex supported as monophyletic [14]. A subsequent analysis by Yan (2019) using combined morphological and molecular data suggested that the *Eupteryx* complex is a paraphyletic group [15].

Because of maternal inheritance, the absence of introns, a high evolutionary rate, and rare recombination, the insect mitochondrial genome has been widely used for phylogenetic and evolutionary analysis [16,17,18,19,20,21], but few mitogenome sequences representative of major lineages of Typhlocybini have been available to facilitate a comprehensive phylogenetic analysis of the tribe. Thus, we assembled and annotated 19 new mitogenomes of typhlocybine, combining them with 48 mitogenome sequences of previously available typhlocybine mitogenomes, to reconstruct phylogenetic relationships between the main lineages to examine the monophyly of Typhlocybini and the phylogenetic status and relationships among generic complexes.

## 2. Materials and Methods

### 2.1. Sample Collection and DNA Extraction

All specimens were stored in vials of 95% ethanol and ultracold (−80 °C) freezers at Northwest A&F University (NWAFU, China) prior to DNA extraction. All specimens were identified based on morphological characters (e.g., venation and male genitalia). Then, total DNA was extracted from the whole body of adult specimens using the EasyPureR Genomic DNA Kit (TransGen, Beijing, China). Information on the collection of all new sequenced specimens is shown in Table S1.

### 2.2. Mitogenome Sequence Analysis

The whole mitogenomes from 19 species were sequenced using the Illumina platform. The quality trimming and assembly of the paired reads were performed using Geneious 8.1.3 with default parameters [22]. The mitochondrial genomes of *Eupteryx minuscula* Lindberg, 1929 (MN910279), *Eurhadina jarrary* Dworakowska, 2002 (MZ014455), *Limassolla lingchuanensis* Chou and Zhang, 1985 (NC_046037), *Typhlocyba* sp. (KY039138), *Yangisunda tiani* Zhang, Huang and Shen, 2003 (MZ014459), and *Zyginella minuta* Yang, 1965 (MT488436), were chosen as reference sequences [11,23,24,25,26]. All 19 complete mitogenomes were annotated in Geneious 8.1.3 [22].

The PCGs were identified through open reading frames and translated into amino acids based on the invertebrate mitochondrial code. The MITOS Web Server was used to identify tRNA genes and predict secondary structures (http://mitos.bioinf.uni-leipzig.de/index.py, accessed on 20 April 2022) [27], and secondary structures were plotted with Adobe Illustrator CS5 manually. Control regions and rRNA genes were identified by the boundary of the adjacent tRNAs (*trnL1* and *trnV*). Tandem repeats in the control regions were predicted using the Tandem Repeats Finder program (http://tandem.bu.edu/trf/trf.html, accessed on 20 April 2022) [28]. The whole-mitogenome map was drawn using CGView (http://stothard.afns.ualberta.ca/cgview_server/, accessed on 25 May 2022) [29]. PhyloSuite v 1.1.15 was used to analyze the base composition, codon usage, composition skewness and relative synonymous codon usage (RSCU) [30]. A sliding-window analysis based on PCGs was used to estimate nucleotide diversity (Pi) using DnaSP v 5.0. in 19 newly sequenced species [31]. Genetic distances and non-synonymous (dN)/synonymous (dS) mutation rates among the 13 PCGs were calculated using MEGA 7.0 and DnaSP v 5.0, respectively [31,32].

### 2.3. Phylogenetic Analysis

Maximum-likelihood analysis and Bayesian inference were performed based on sixty-seven species of Typhlocybinae selected as the ingroup, including two species of Alebrini, four species of Empoascini, five species of Dikraneurini, five species of Erythroneurini, thirty-four species of Typhlocybini and seventeen species of Zyginellini [11,24,25,26,33,34,35,36,37,38,39]. *Scaphoideus varius* Vilbaste, 1968 (Deltocephalinae), and *Evacanthus heimianus* Kuoh, 1981 (Evacanthinae), were chosen as outgroups [40]. All sixty-nine mitochondrial genomes were deposited in GenBank (Table 1).

Alignment of the 13 PCGs and 2 rRNAs was performed using the MAFFT plugin within PhyloSuite [30] and the MAFFT online service [41], respectively (the PCGs and AA with G-INS-i strategy and two rRNAs with Q-INS-i strategy). Gblocks was used to remove ambiguous sites and gaps [42], and then the results were concatenated in PhyloSuite [30]. The optimal partition schemes and substitution models were obtained by using PartitionFinder2 [43] (Tables S2 and S3). The “greedy” algorithm was used with branch lengths estimated as “linked” and the Bayesian information criterion [30].

Five datasets were assembled for phylogenetic analysis: the PCG12 matrix (protein-coding genes with the first and second codon positions); the PCG12R matrix (protein-coding genes with the first and second codon positions, the 2 rRNA genes); the PCG123 matrix (protein-coding genes with all codon positions); the PCG123R matrix (protein-coding genes with all codon positions, the 2 rRNA genes); and the AA matrix (amino acid sequences of PCGs).

Phylogenetic analyses were conducted using maximum likelihood (ML) and Bayesian inference (BI). The maximum likelihood (ML) and Bayesian inference (BI) were conducted using IQ-TREE [44] and MrBayes 3.2.6 [45], respectively. The ML tree was constructed with the ML + rapid bootstrap algorithm (1000 replicates). In the Bayesian analysis, the default settings were used with 5 × 10^6^ MCMC generations after reaching stationarity (average standard deviation of split frequencies < 0.01), with estimated sample size > 200 and potential scale reduction factor ≈ 1 [45].

## 3. Results

### 3.1. Mitogenome Organization and Gene Content

**Figure 1:**
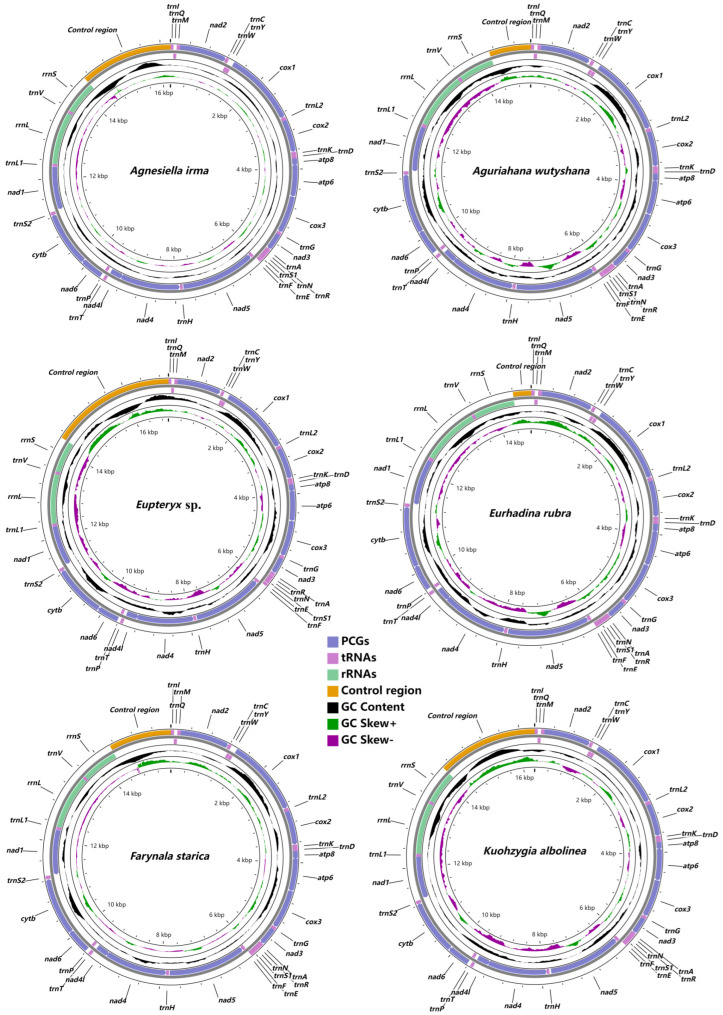
The mitogenomes of *Agnesiella irma*, *Aguriahana wutyshana*, *Eupteryx* sp., *Eurhadina rubra*, *Farynala starica* and *Kuohzygia albolinea*.

The genome size, composition and organization of the 19 newly sequenced mitogenomes are summarized in Table S4 and Figure 1 and Figures S1–S3. The total length ranged from 14,749 bp to 17,220 bp. This variation in size is mainly due to the variable size of the control region. All 19 newly sequenced mitogenomes encode 37 genes: 13 protein-coding genes, 2 ribosomal RNA genes, 22 transfer RNA genes and a control region (Figure 1 and Figures S1–S3. All genes in 19 newly sequenced mitogenomes have the same arrangement as the ancestral insect mitogenomes [46].

A heavy AT nucleotide bias was observed in the 19 new mitogenomes, with A + T content ranging from 75.3% to 79.3% (Table S4), which is similar to previous typhlocybine mitogenomes. The rRNAs have the highest AT content, followed by tRNAs; the PCGs have the lowest AT content.

### 3.2. Protein-Coding Genes and Codon Usage

**Figure 2:**
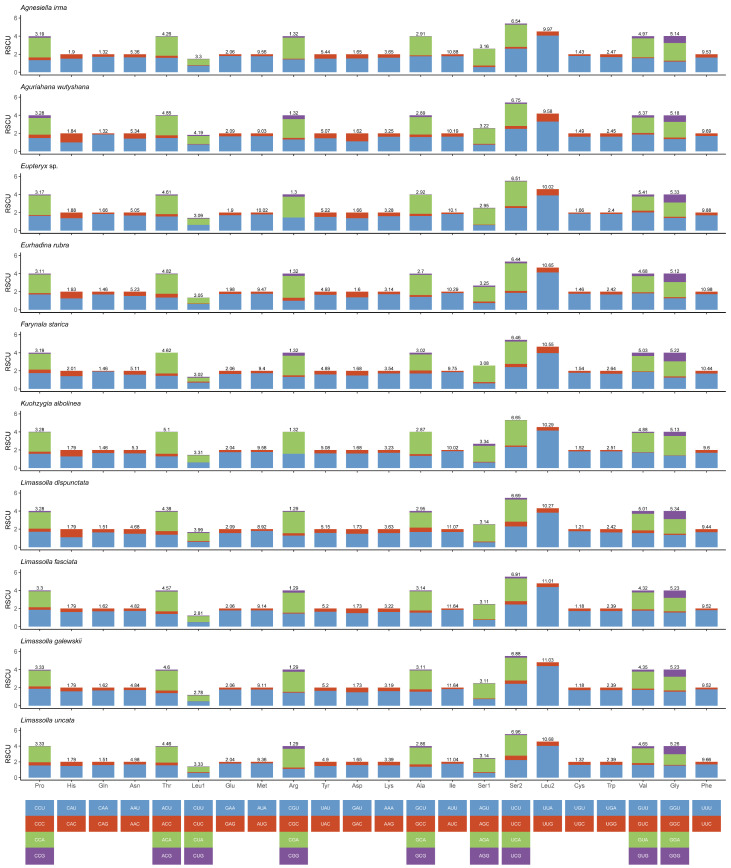
Relative synonymous codon usage (RSCU) in the mitogenomes of *Agnesiella irma*, *Aguriahana wutyshana*, *Eupteryx* sp., *Eurhadina rubra*, *Farynala starica*, *Kuohzygia albolinea*, *Limassolla dispunctata*, *Limassolla fasciata*, *Limassolla galewskii* and *Limassolla uncata*.

**Figure 3:**
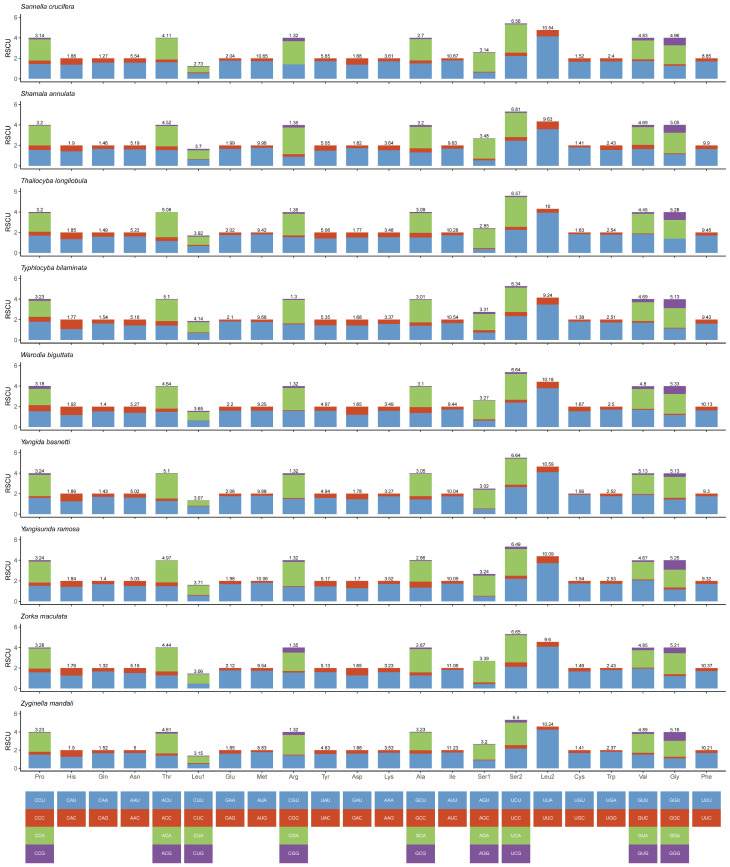
Relative synonymous codon usage (RSCU) in the mitogenomes of *Sannella crucifera*, *Shamala annulata*, *Thailocyba longilobula*, *Typhlocyba bilaminata*, *Warodia biguttata*, *Yangida basnetti*, *Yangisunda ramosa*, *Zorka maculata* and *Zyginella mandali*.

The total size of the 13 PCGs of 19 mitogenomes ranged between 10,884 bp (*Thailocyba longilobula*) and 10,962 bp (*Yangida basnetti*). In 13 protein-coding genes, the third codon position has the highest AT content, and the second codon position has the lowest AT content (Table S4). Most of the PCGs in the 19 new mitogenomes started with the codon ATN, but *nad5* starts with TTG in *Eupteryx* sp., and *atp8* starts with TTG, except in *Zorka maculata* and *Thailocyba longilobula* (Table S5). Most PCGs of the 19 new mitogenomes stop with TAA or TAG, while the incomplete stop codon T is used in some mitogenomes (*cox1*, *cox2*, *cox3*, *nad4*, *nad5* and *nad6*). Truncated termination codons might be converted into TAA through polyadenylation [47]. In all 19 sequenced mitogenomes, the frequency of use of ATG and ATT codons is higher than those of other start codons, while the TTG frequency is the lowest. The frequency of the TAA stop codon is higher than that of a single T, and TAG codon usage is the lowest (Table S5). The relative synonymous codon usage (RSCU) for 19 complete mitogenomes is shown in Figure 2 and Figure 3. The analysis shows that the codon usage has a heavy AT nucleotide bias. The most frequently used codons were UUA (Leu2), UCU (Ser2), AUU (Ile), UUU (Phe) and AUA (Met). Additionally, the codons Thr (ACG), Ser1 (AGG), Gly (GGC), Leu1 (CUC) and Arg (CGC) were not found in some mitogenomes among the 19 species.

### 3.3. Transfer and Ribosomal RNA Genes

In the 19 sequenced mitogenomes, 22 tRNAs and 2 rRNA genes were identified. The total lengths of 22 tRNAs ranged from 1420 bp (*Limassolla dispunctata*) to 1455 bp (*Zyginella mandali*). The AT and GC skew values in tRNAs of these 19 mitogenomes were positive, except for *Zyginella mandali* and *Limassolla uncata*, which show a negative AT skew (Table S4). The secondary structures of the 22 tRNAs are shown in Figures S4–S22. These tRNAs can be folded into the typical cloverleaf structure, except for *trnS1*, with a reduced DHU arm that forms a simple loop in *Agnesiella ramo*, *Eupteryx* sp., *Limassolla dispunctata*, *Limassolla fasciata*, *Limassolla galewskii*, *Limassolla uncata*, *Sannella crucifera*, *Typhlocyba bilaminata*, *Yangida basnetti* and *Yangisunda ramose*; the lack of the DHU arm in *trnS1* has been observed in most metazoans [48]. The TΨC of *trnV* arms was missing in *Farynala starica.* The mismatched types of unpaired bases in these 19 new mitogenomes were A-A, U-U, G-G, A-C, A-G, U-C and G-U.

The large RNA genes (*rrnL*) were located between *trnL1* and *trnV*, and the small ribosomal RNA genes (*rrnS*) were located between *trnV* and the control region. The total lengths of 2 rRNAs ranged from 1712 bp (*Zorka maculata*) to 2014 bp (*Eurhadina rubra*), and the 2 rRNAs in these 19 mitogenomes showed a negative AT skew and a positive GC skew (Table S4).

### 3.4. Control Region

**Figure 4:**
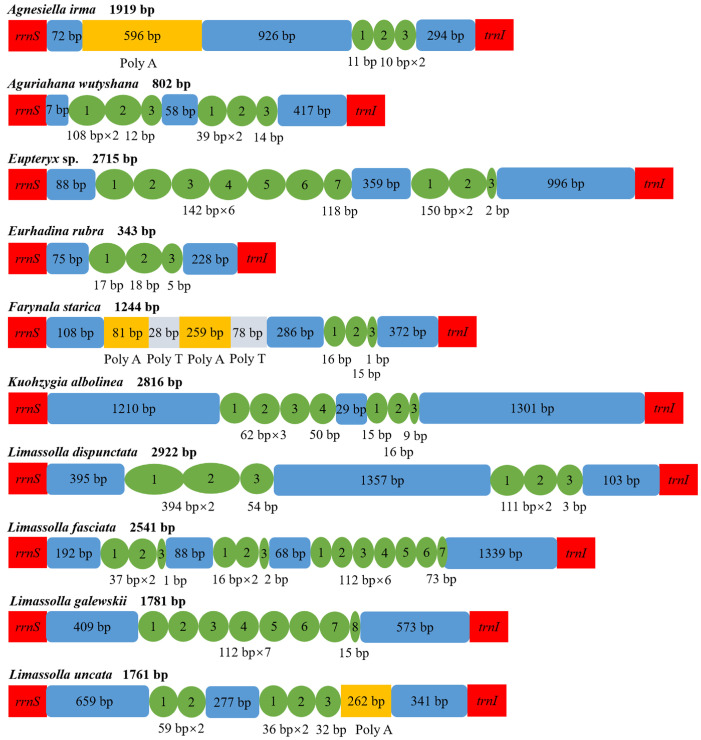
Control regions in the mitochondrial genomes of *Agnesiella irma*, *Aguriahana wutyshana*, *Eupteryx* sp., *Eurhadina rubra*, *Farynala starica*, *Kuohzygia albolinea*, *Limassolla dispunctata*, *Limassolla fasciata*, *Limassolla galewskii* and *Limassolla uncata*. The green circles indicate the repeat units; the blue boxes indicate the non-repeat regions; the yellow and gray boxes indicate poly (A) and poly (T), respectively.

The control region is also called the major non-coding region or the A + T-rich region, in which the sequence composition and copy numbers of repeat units are quite variable (Figure 4 and Figure S23). The control region varies in size from 343 bp (*Eurhadina rubra*) to 2922 bp (*Limassolla dispunctata*), with AT content ranging from 63.7% (*Kuohzygia albolinea*) to 89.8% (*Farynala starica*) among these 19 mitogenomes.

Among the control regions of the 19 newly sequenced mitogenomes, all had tandem repeats, except for *Zorka maculata*. *Agnesiella irma*, *Eurhadina rubra*, *Farynala starica*, *Limassolla galewskii*, *Shamala annulata*, *Typhlocyba bilaminata* and *Zyginella mandali* had one tandem repeat region. *Aguriahana wutyshana*, *Eupteryx* sp., *Kuohzygia albolinea*, *Limassolla dispunctata*, *Limassolla uncata*, *Sannella crucifera*, *Warodia biguttata*, *Yangida basnetti* and *Yangisunda ramosa* had two tandem repeat regions; *Limassolla fasciata* and *Thailocyba longilobula* had three tandem repeat regions. In some species, poly A or poly T was observed.

### 3.5. Nucleotide Diversity and Evolutionary Rate Analysis

**Figure 5:**
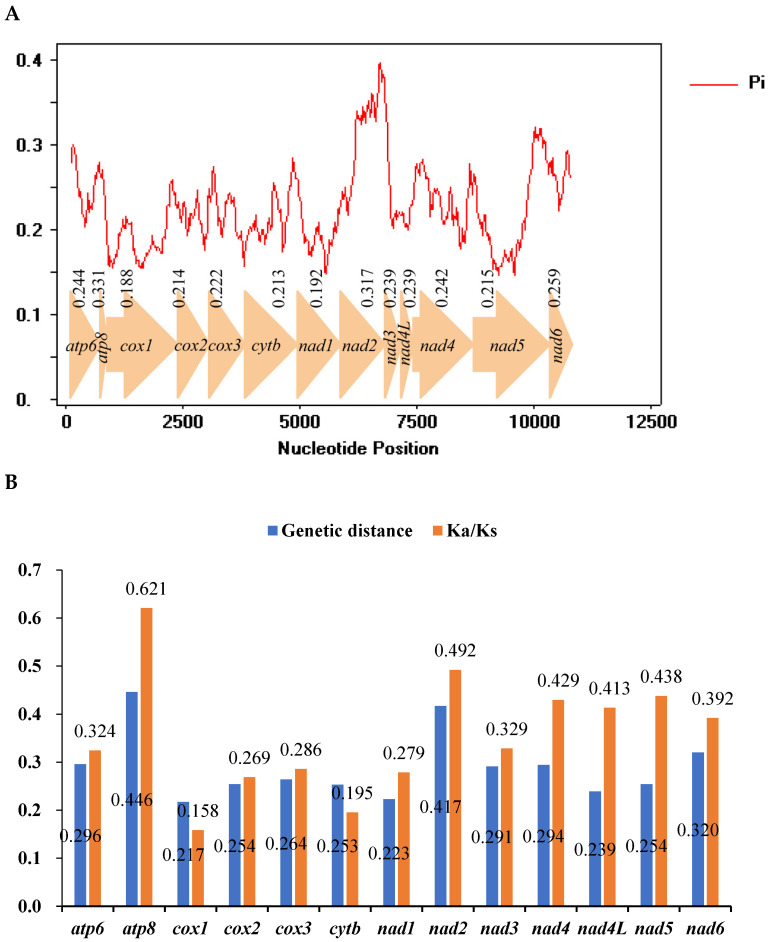
(**A**) Sliding-window analyses based on 13 PCGs of 19 mitogenomes. The red line represents the value of nucleotide diversity (Pi) (200 bp window with 20 bp step). (**B**) Average genetic distance and non-synonymous (Ka) to synonymous (Ks) substitution rates of 13 PCGs of 19 mitogenomes.

Nucleotide diversity based on 13 PCGs from the 19 sequenced species (Figure 5A) ranges from 0.188 (*cox1*) to 0.331 (*atp8*). Comparing the 13 PCGs, *atp8*, *nad2* and *nad6* show high variability. In contrast, *cox1* and *nad1* are comparatively conserved genes. The evolutionary rate can be estimated based on average non-synonymous (Ka) and synonymous (Ks) substitution rates [49]. The average Ka/Ks rates for the 13 PCGs ranged from 0.158 to 0.621, which is less than 1, indicating the existence of purifying selection for these genes (Figure 5B). The genes *atp8*, *nad5* and *nad2* have high Ka/Ks ratios compared to the comparatively low values for *cox1*, *cytb* and *cox2* (Figure 5B). Low Ka/Ks values indicate strong purifying selection. Pairwise genetic distances among these 19 mitogenomes show that *atp8*, *nad2* and *nad6* evolve comparatively fast, while *cox1*, *nad1* and *nad4L* evolve relatively slowly (Figure 5B).

The gene *cox1* is commonly used as a marker for species identification [50,51]; it presented the lowest nucleotide diversity (Pi), Ka/Ks rates and pairwise genetic distances in our analysis. These results are consistent with previous studies of Typhlocybinae [11].

### 3.6. Phylogenetic Relationships

**Figure 6:**
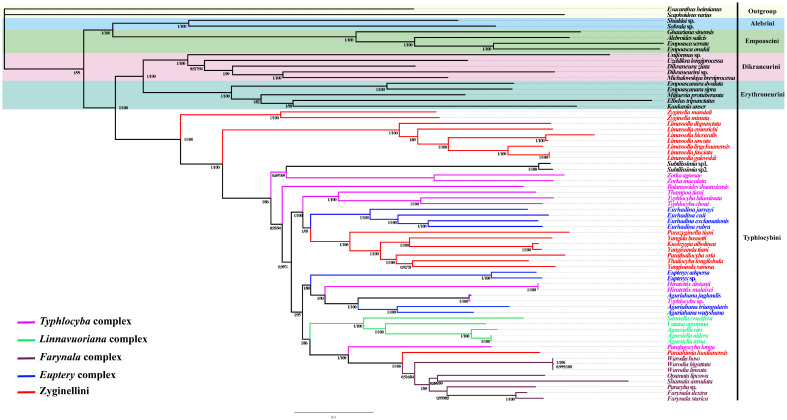
The phylogenetic tree generated by BI and ML analyses based on PCG123R dataset. The numbers are Bayesian posterior probabilities and bootstrap support values.

**Figure 7:**
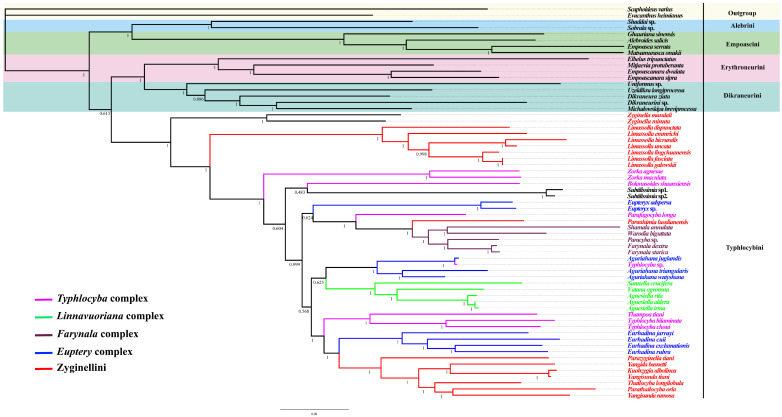
The phylogenetic tree generated by BI analysis based on AA dataset. The numbers are Bayesian posterior probabilities.

Highly consistent phylogenetic topologies based on the five datasets (PCG12, PCG123, PCG12R, PCG123R and AA) were generated, with most branches receiving maximum support in both BI and ML trees (Figure 6, Figure 7 and Figures S24–S30), but the AA topology based on the BI method differs slightly, as shown in Figure 7. Individual clade nodes with Bayesian posterior probabilities and bootstrap scores. The best models are listed in Tables S2 and S3. The results (Figure 6) supported the monophyly of Typhlocybinae. Furthermore, the recovery of Alebrini, Dikraneurini, Empoascini and Erythroneurini as monophyletic clades is consistent with Lin et al. (2021) [11]. And Alebrini was recovered as sister to Empoascini, and Dikraneurini was recovered as sister to Erythroneurini, both with strong support.

The clade with all of the members of Typhlocybini and Zyginellini has high support (PP = 1, BS = 100), but the latter, as traditionally defined (with hindwing veins CuA apparently directly connected to MP), was not recovered as monophyletic. Clades comprising two species of *Zyginella* and seven species of *Limassolla* were successive sister groups to the rest of the species, including Typhlocybini (sensu stricto) and some additional genera and species of Zyginellini (PP = 1, BS = 100). Except for *Paraahimia luodianensis*, the other seven species of Zyginellini (*Parazyginella tiani*, *Yangida basnetti*, *Kuohzygia albolinea*, *Yangisunda tiani*, *Parathailocyba orla*, *Thailocyba longilobula*, *Yangisunda ramosa*) formed a well-supported clade (PP = 1, BS = 100). In the AA dataset based on BI analyses, the seven species of Zyginellini in the most extreme position form a sister group to the genus *Eurhadina*. The overall relationships are congruent with those of Cao et al. (2023), but the present dataset includes several genera and species not included in the former study [12].

Within Typhlocybini, genera for which multiple representatives are included are recovered as monophyletic, except for the paraphyletic typhlocybine genera *Yangisunda* and *Typhlocyba*. *Warodia*, *Farynala*, *Opamata*, *Paracyba* and *Shamala* form a clade, supporting the monophyly of the *Farynala* complex, consistent with Huang (2003) [14]. In the AA dataset based on BI analyses, *Farynala complex* is a sister group to the genus *Eupteryx. Sannella*, *Vatana* and *Agnesiella* also form a clade (PP = 1, BS = 100), supporting the monophyly of the *Linnavuoriana* complex. *Eupteryx*, *Aguriahana* and *Eurhadina* are morphologically similar in having three cross veins on the hindwing, but these genera did not cluster together; thus, the *Eupteryx* complex is paraphyletic. *Bolanusoides*, *Zorka*, *Typhlocyba*, *Thampoa* and *Hiratettix*, placed in the *Typhlocyba* complex, also failed to form a clade. Different from the topologies of the PCG123R dataset, *Zorka* does not form a sister group to *Subtilissimia* in the PCG12 dataset based on BI analyses. And in the topologies of phylogenetic trees based on PCG12R, the genus *Subtilissimia* is divided first, showing a sister group to the remaining Zyginellini, except *Limassolla* and *Zyginella*.

## 4. Discussion

### 4.1. Comparative Analysis of Typhlocybine Mitogenomes

The 19 sequenced typhlocybine mitogenomes were conservative and similar to other species of Typhlocybinae from previous studies [11,23,24,25,26,33,34,35,36,37,38,39]. The variation in the size of the total length is mainly due to the variable size of the control region, as the sequence composition and copy numbers of repeat units in the control region are quite variable. Similar to previous typhlocybine mitogenomes, a heavy AT nucleotide bias was observed in the 19 new mitogenomes, and the rRNAs have the highest AT content, followed by tRNAs; the PCGs have the lowest AT content [11].

All tRNAs could be folded into the typical cloverleaf structure, except for *trnS1*, with a reduced DHU arm in some species; the lack of the DHU arm in *trnS1* has been observed in most metazoans [48]. The TΨC of *trnV* arms was missing in *Farynala starica*, and unpaired bases are also found in *trnQ* [11]. We found seven types of mismatched bases; these mismatched base pairs have been reported in previous mitogenomes of Typhlocybinae [11].

Mitochondrial genes have been widely used as genetic markers, especially the *cox1* gene [50,51]. However, *cox1* presented the lowest nucleotide diversity (Pi), Ka/Ks rates and pairwise genetic distances in our analysis, consistent with Lin et al. (2021) [11]. The gene *atp8* may be too short to be phylogenetically informative; *nad2* and *nad6* could be selected as more suitable DNA markers to clarify the phylogenetic relationships of closely related species of Typhlocybinae.

### 4.2. The Monophyly of Typhlocybini

Typhlocybini was first proposed by Kirschbaum (1868) [52]; however, the classification and composition have long been controversial, with different scholars adopting alternative definitions of the group. In his monograph on Rhynchota of British India, Distant (1908) placed *Typhlocyba*, *Eupteryx* and *Motschulskia* (the last one now included in Dikraneurini) in his “Typhlocybaria” [53]. In a subsequent study of world Typhlocybinae, McAtee (1934) divided Typhlocybini into Eupterygini and Jorumini on the basis of the presence or absence of closed apical cells in the hindwing [54]. Young (1952) separated Erythroneurini from Typhlocybini based on the unbranched hindwing anal vein of the former group but retained the broad concept of Typhlocybini [55]. Mahmood and Ahmed (1968) separated Empoascini from Typhlocybini based on the absence of closed apical cells in the latter [56]; Dworakowska (1977) established the tribe Zyginellini on the basis of the presence of a single transverse vein on the hindwing and CuA directly connected to MP [10]. Alternatively, Anufriev (1978) split Typhlocybini (sensu stricto) into two subtribes, i.e., Typhlocybina and Eupterygina, based on the presence or absence of vein R + M in the hindwing [57]. Most authors subsequently followed the classification of Dworakowska (1979), whose system of six tribes was mainly based on venation [9].

Some more recent authors questioned the status of the tribe Zyginellini, considering the single hindwing character traditionally used to recognize the tribe to be inadequate. Taking into account the characteristics of the forewing venation, male genitalia and molecular data, several scholars suggested that Zyginellini should be merged with Typhlocybini [2,3,11,58]. However, the recent molecular phylogeny presented by Lu et al. (2021) recovered Zyginellini as sister to Typhlocybini [13]. None of the above studies included extensive samples of Typhlocybini and Zyginellini, which may account for their conflicting results.

To provide a much more robust estimate of relationships within Typhlocybini (sensu lato), we analyzed a much larger sample of Typhlocybini (sensu lato) compared to previous studies, with our dataset comprising 67 mitogenome sequences, including 10 new Typhlocybini mitogenomes from 10 genera and 9 new Zyginellini mitogenomes from 6 genera. Analyses of these data yielded phylogenetic estimates that were largely consistent among datasets and analyses, with most branches receiving strong support. The results show that Typhlocybini and Zyginellini together form a monophyletic sister group to a clade comprising Dikraneurini + Erythroneurini, consistent with the phylogenomic analysis by Cao et al. (2023) [12]. However, also consistent with Cao et al. (2023), our results show that Zyginellini (sensu Dworakowska) is polyphyletic, with different zyginelline genera placed in a paraphyletic grade subtending Typhlocybini and in two additional independent branches derived from within Typhlocybini. Although the taxon sample in our study only partially overlaps that of Cao et al. (2023), the topologies are congruent [12]. Our study included several genera not included by Cao et al. (2023) and provides further evidence that the hindwing trait traditionally used to define Zyginellini arose independently at least three times during the evolution of Typhlocybini [12]. These results confirm that Zyginellini should be treated as a junior synonym of Typhlocybini.

### 4.3. The Definition of Genera Complexes in Typhlocybini

The hindwing venation includes the most important taxonomic characteristics traditionally used to define tribes of Typhlocybinae. The tribe Typhlocybini (sensu lato) exhibits greater variation in hindwing venation than most other typhlocybine tribes, and this has been a source of confusion for previous taxonomists. According to the number of cross veins on the hindwing, the genera of Typhlocybini can be classified into three informal groups: (1) the group with three cross veins (Eupterygini, one of the junior synonyms of Typhlocybini; Eupterygina); (2) the group with two cross veins (Typhlocybini sensu stricto; Typhlocybina); and (3) the group with one cross vein (Zyginellini). Within Typhlocybini sensu stricto, four informal complexes, i.e., the *Farynala* complex, *Eupteryx* complex, *Typhlocyba* complex and *Linnavuoriana* complex, were previously proposed for the Old World genera [59]. Most of the endemic Neotropical genera of Typhlocybini exhibit their own unique hindwing venational patterns and represent a separate, plesiomorphic lineage sister to the Old World members of this tribe [3,12].

The *Farynala* complex is characterized by pygofer with several macrosetae, the subgenital plate with a long macroseta basally, the paramere without subapical tooth and the connective developed and laminate. Huang (2003) included five genera in this complex: *Farynala*, *Opamata*, *Shamala*, *Warodia* and *Paracyba*. The monophyly of this group was supported by Yan (2019) [14,15]. In our study, these five genera also form a clade with strong support, confirming the monophyly of this complex.

Some previous authors (e.g., McAtee 1936) considered “Eupterygini” to be a separate tribe or a subtribe (Anufriev 1978), but it is more recently considered to be the *Eupteryx* complex within Typhlocybini, recognized for having three cross veins on the hindwing [60,61]. Our analyses failed to recover the *Eupteryx* complex as monophyletic, placing the three included genera, *Eurhadina*, *Eupteryx* and *Aguriahana*, on independent branches. This result is also consistent with those of Yan (2019) [15] and Cao et al. (2023) [12]. *Eurhadina* was recovered as sister to one clade of “Zyginellini” (excepting genera *Limassolla*, *Paraahimia* and *Zyginella*) with strong support, consistent with the shared morphological characters of the subgenital plate with a long macrosetae basally and paramere without a subapical tooth. The other two genera, *Eupteryx* and *Aguriahana*, are grouped with *Hiratettix*, forming a clade comprising taxa in which the subgenital plate has more than one long macroseta basally and the aedeagus has well-developed processes.

*Bolanusoides*, *Zorka*, *Typhlocyba*, *Thampoa* and *Hiratettix* in the *Typhlocyba* complex were not recovered as a monophyletic group, consistent with the morphology-based analysis by Huang (2003), who recovered this complex as paraphyletic [14]. *Typhlocyba* and *Thampoa* always form a clade, also in agreement with Huang (2003) [14]. The *Typhlocyba* complex included genera with two cross veins on the hindwing and a single basal macroseta on the subgenital plate, but genera sharing these traits may otherwise be distinctly different in appearance. Thus, other morphological characters need to be studied to help redefine the complex.

The *Linnavuoriana* complex is characterized by a slender body, a pygofer with a posterior process or protrusion, a subgenital plate without a long macroseta, a paramere with a subapical tooth, an aedeagus with a well-developed dorsal apodeme and a long aedeagal shaft with a simple process. Huang (2003) included four genera in this complex, *Vatana*, *Agnesiella*, *Linnavuoriana* and *Amurta* [14]. In our study, *Vatana* and *Agnesiella* are sister to *Sannella*, forming a clade consistent with the *Linnavuoriana* complex. *Sannella* appears to be related to Zyginellini due to its hindwing having the submarginal vein and CuA vagualy connected by a transverse vein [14,62]. However, in our ML and BI analyses, *Sannella* is sister to genera of the *Linnavuoriana* complex, and based on the consistent morphological characteristics, we suggest assigning it to the *Linnavuoriana* complex.

Our analyses show that some generic groups previously recognized based on morphological characters (e.g., the *Farynala* and *Linnavuoriana* complexes) correspond to monophyletic lineages, but others do not. This suggests that the morphological variation within Typhlocybini needs to be re-assessed more fully to identify characters that reliably diagnose monophyletic groups. Our present results, based on analyses of complete mitogenome sequences, are highly consistent with those obtained from the analysis of large numbers of nuclear gene loci [12]. This indicates that analyses of mitogenome sequences provide a reliable alternative to more extensive phylogenomic analyses of nuclear gene datasets for resolving relationships among genera and species of Typhlocybini. Further studies incorporating additional genera and species, including representatives of other regional faunas, are needed to provide a more complete assessment of relationships within this tribe.

## Figures and Tables

**Table 1:** The sequences used in this study.

Subfamily	Tribe	Species	Accession Number
Evacanthinae		*Evacanthus heimianus*	MG813486
Deltocephalinae		*Scaphoideus varius*	KY817245
Typhlocybinae	Alebrini	*Shaddai* sp.	MZ014457
		*Sobrala* sp.	MZ014458
	Empoascini	*Ghauriana sinensis*	MN699874
		*Alebroides salicis*	MZ014449
		*Empoasca serrata*	MZ014453
		*Empoasca onukii*	NC_037210
	Dikraneurini	*Dikraneura zlata*	MZ014450
		*Dikraneurini* sp.	MZ014451
		*Uniformus* sp.	MW272457
		*Uzeldikra longiprocessa*	MW284821
		*Michalowskiya breviprocessa*	MW264489
	Erythroneurini	*Elbelus tripunctatus*	MZ014452
		*Kaukania anser*	MZ014456
		*Mitjaevia protuberanta*	NC_047465
		*Empoascanara dwalata*	MT350235
		*Empoascanara sipra*	MN604278
	Typhlocybini	*Agnesiella aldera*	MW284835
		*Agnesiella irma*	ON022031
		*Agnesiella rita*	MW284822
		*Aguriahana wutyshana*	ON000924
		*Aguriahana juglandis*	MW284823
		*Aguriahana triangularis*	MW284824
		*Bolanusoides shaanxiensis*	MN661136
		*Eupteryx adspersa*	MZ014454
		*Eupteryx* sp.	ON022032
		*Eurhadina cuii*	MW284836
		*Eurhadina exclamationis*	MW284837
		*Eurhadina rubra*	ON022033
		*Eurhadina jarrary*	MZ014455
		*Farynala dextra*	MW284838
		*Farynala starica*	ON022034
		*Hiratettix distanti*	MW284839
		*Hiratettix malaisei*	MW284840
		*Opamata lipcowa*	MW284842
		*Paracyba* sp.	MW284842
		*Parafagocyba longa*	MW284825
		*Sannella crucifera*	OL960659
		*Shamala annulata*	ON022035
		*Subtilissimia* sp1.	MW284826
		*Subtilissimia* sp2.	MW284827
		*Thampoa tiani*	MW284828
		*Typhlocyba bilaminata*	ON022036
		*Typhlocyba choui*	MW284829
		*Typhlocyba* sp.	KY039138
		*Vatana ogromna*	MW284830
		*Warodia biguttata*	ON022037
		*Warodia hoso*	MW284831
		*Warodia lineata*	MW284832
		*Zorka agnesae*	MW284834
		*Zorka maculata*	ON022038
	Zyginellini	*Kuohzygia albolinea*	OL960654
		*Limassolla lingchuanensis*	NC_046037
		*Limassolla bicruralis*	MT683892
		*Limassolla dispunctata*	OL960655
		*Limassolla emmrichi*	MW272458
		*Limassolla fasciata*	OL960656
		*Limassolla galewskii*	OL960657
		*Limassolla uncata*	OL960654
		*Paraahimia luodianensis*	NC_047464
		*Parathailocyba orla*	MN894531
		*Parazyginella tiani*	MT683891
		*Thailocyba longilobula*	OL960660
		*Yangida basnetti*	ON000925
		*Yangisunda ramosa*	OL960661
		*Yangisunda tiani*	MZ014459
		*Zyginella mandali*	ON055365
		*Zyginella minuta*	MT488436

## Data Availability

The data that support the findings of this study are openly available in the National Center for Biotechnology Information at https://www.ncbi.nlm.nih.gov/nuccore (accessed on 19 March 2022).

## Supplementary Materials

The following supporting information can be downloaded at https://www.mdpi.com/article/10.3390/insects14110842/s1: Table S1. Collected information on all species in this study; Tables S2 and S3. The best-partitioning models for maximum-likelihood (ML) and Bayesian inference (BI) methods based on five datasets selected by PartitionFinder; Table S4. Nucleotide composition of the mitogenomes of 19 newly sequenced species. Table S5. Start and stop codon usage in the 19 mitochondrial genomes; Figure S1. Organization of *Limassolla dispunctata*, *Limassolla fasciata*, *Limassolla galewskii*, *Limassolla uncata*, *Sannella crucifera* and *Shamala annulate*; Figure S2. Organization of the mitochondrial genome of *Thailocyba longilobula*, *Typhlocyba bilaminata*, *Warodia biguttata*, *Yangida basnetti*, *Yangisunda ramosa* and *Zorka maculate*; Figure S3. Organization of the mitochondrial genome of *Zyginella mandali*; Figures S4–S22. Prediction of secondary structures for the tRNAs of *Agnesiella irma*; *Aguriahana wutyshana*; *Eupteryx* sp.; *Eurhadina rubra*; *Farynala starica*; *Kuohzygia albolinea*; *Limassolla dispunctata*; *Limassolla fasciata*; *Limassolla galewskii*; *Limassolla uncata*; *Sannella crucifera*; *Shamala annulata*; *Thailocyba longilobula*; *Typhlocyba bilaminata*; *Warodia biguttata*; *Yangida basnetti*; *Yangisunda ramosa*; *Zorka maculata*; *Zyginella mandali*; Figure S23. Structures of the control regions of *Sannella crucifera*, *Shamala annulata*, *Thailocyba longilobula*, *Typhlocyba bilaminata*, *Warodia biguttata*, *Yangida basnetti*, *Yangisunda ramosa*, *Zorka maculata* and *Zyginella mandali*; Figures S24 and S25. BI and ML trees based on PCG12; Figures S26 and S27. BI and ML trees based on PCG12R; Figures S28 and S29. BI and ML trees based on PCG123; Figure S30. ML tree based on AA.
