# Supplementary material for: Investigating Monophyly of Typhlocybini Based on Complete Mitochondrial Genomes with Characterization and Comparative Analysis of 19 Species (Hemiptera: Cicadellidae: Typhlocybinae)
Source: Insects. 2023 Oct 30;14(11):842. doi: 10.3390/insects14110842 (PMC10671860; doi:10.3390/insects14110842)
Supplement: Supplementary file 1 [file insects-14-00842-s001.zip › Supplementary Tables S1-S5.pdf]

Table S1. Collect information on all species in this study.

Species	Locality	Date	Collector
Agnesiella irma	West Mountain, Kunming City, Yunnan Province, China	26-Jun-2021	Xian Zhou
Aguriahana wutyshana	Shoufeng Mountain, Baoji City, Shaanxi Province, China	23-May-2021	Juan Zhou
Eupteryx sp.	Gengda Town, Wenchuan County, Sichuan Province, China	17-Jul-2021	Junjie Wang
Eurhadina rubra	Tongmu village, Wuyishan City, Fujian Province, China	17-Jul-2021	Juan Zhou
Farynala starica	Xiaojie Town, Wenshan City, Yunnan Province, China	17-Jul-2021	Xian Zhou
Kuohzygia albolinea	Guanshan, Yichun City, Jiangxi Province, China	17-Jul-2018	Chen Yang
Limassolla dispunctata	Yangling, Shaanxi Province, China	23-May-2021	Xian Zhou
Limassolla fasciata	Chebaling National Nature Reserve,Guangdong Province, China	18-Jul-2020	Junjie Wang
Limassolla galewskii	Jiangkou County,Tongren City, Guizhoui Province, China	31-Aug-2020	Xian Zhou
Limassolla unctata	Nanling,Guangdong Province, China	23-Jul-2020	Junjie Wang
Sannella crucifera	Hongfeng Forest Park, Debao County,Guangxi Province, China	21-Jul-2021	Xian Zhou
Shamala annulata	Hongfeng Forest Park, Debao County,Guangxi Province, China	21-Jul-2021	Xian Zhou
Thailocyba longilobula	Huanglian Mountain, Luchun County, Yunnan Province, China	13-Jul-2021	Xian Zhou
Typhlocyba bilaminata	Jiuxiang, Yichang County, Yunnan Province, China	8-Jul-2021	Yulin Hu
Warodia biguttata	Guzhang Town, Tianlin County, Guangxi Province, China	19-Jul-2021	Xian Zhou
Yangida basnetti	Sun-River Forest Park, Pu'er City, Yunnan Province, China	2-Jul-2021	Xian Zhou
Yangisunda ramosa	Langping Town, Tianlin County, Baise City, Guangxi Province, China	24-Jul-2021	Xian Zhou
Zorka maculata	Baishi Town, Zhong County, Chongqing City, China	18-Jul-2019	Shuanghu Lin
Zyginella mandali	Beida village, Shilin Town, Kunming City, Yunnan Province, China	10-Jul-2021	Xian Zhou

Table S2. The best partitioning schemes and models for Maximum likelihood (ML) method based on four datasets selected by PartitionFinder.

Dataset	Models	Partitioning scheme
PCG12	GTR+I+G	*atp6*, *nad3*
	GTR+I+G	*atp8*, *nad6*, *nad2*
	GTR+I+G	*cytb*, *cox1*
	TIM+I+G	*cox3*, *cox2*
	K81UF+I+G	*nad1*
	TVM+I+G	*nad4L*, *nad5*, *nad4*
PCG12R	GTR+I+G	*atp6*, *nad3*
	GTR+I+G	*nad6*, *atp8*, *nad2*
	GTR+I+G	*cytb*, *cox1*
	TIM+I+G	*cox3*, *cox2*
	K81UF+I+G	*nad1*
	TVM+I+G	*nad4L*, *nad5*, *nad4*
	TVM+I+G	*rrnS, rrnL*
PCG123	GTR+I+G	*atp6*_codon1, *nad3*_codon1, *nad6*_codon1
	TVM+I+G	*atp6*_codon2, *nad3*_codon2, *nad6*_codon2, *atp8*_codon2, *nad2*_codon2
	TRN+I+G	*atp6*_codon3, *atp8*_codon3, *nad6*_codon3
	TRN+I+G	*nad2*_codon1, *atp8*_codon1
	GTR+I+G	*cytb*_codon1, *cox1*_codon1
	TVM+I+G	*cox3*_codon2, *cox2*_codon2, *cytb*_codon2, *cox1*_codon2
	TVM+I+G	*cox1*_codon3, *nad3*_codon3, *cox3*_codon3, *cox2*_codon3
	GTR+I+G	*cox2*_codon1, *cox3*_codon1
	TRN+G	*cytb*_codon3
	TVM+I+G	*nad1*_codon1
	GTR+I+G	*nad5*_codon2, *nad4*_codon2, *nad1*_codon2, *nad4L*_codon2
	HKY+I+G	*nad4*_codon3, *nad1*_codon3, *nad4L*_codon3, *nad5*_codon3
	TRN+G	*nad2*_codon3
	GTR+I+G	*nad5*_codon1, *nad4L*_codon1, *nad4*_codon1
PCG123R	GTR+I+G	*atp6*_codon1, *nad3*_codon1, *nad6_codon1*
	TVM+I+G	*atp6*_codon2, *cox3*_codon2, *cox2*_codon2, *cytb*_codon2, *cox1*_codon2
	TRN+I+G	*atp6*_codon3, *atp8*_codon3, *nad6*_codon3
	TRN+I+G	*nad2*_codon1, *atp8*_codon1
	TVM+I+G	*atp8*_codon2, *nad2*_codon2, *nad3*_codon2, *nad6*_codon2
	GTR+I+G	*cytb*_codon1, *cox1*_codon1
	TVM+I+G	*cox1*_codon3, *nad3*_codon3, *cox3*_codon3, *cox2*_codon3
	GTR+I+G	*cox2*_codon1, *cox3*_codon1
	TRN+G	*cytb*_codon3
	TVM+I+G	*nad1*_codon1
	GTR+I+G	*nad5*_codon2, *nad4*_codon2, *nad1*_codon2, *nad4L*_codon2
	HKY+I+G	*nad4*_codon3, *nad1*_codon3, *nad4L*_codon3, *nad5*_codon3
	TRN+G	*nad2*_codon3
	GTR+I+G	*nad5*_codon1, *nad4L*_codon1, *nad4*_codon1
	TVM+I+G	*rrnL*, *rrnS*
AA	MTART+I+G+F	*atp8*, *nad2*, *cox2*, *atp6*, *nad3*, *cox3*, *nad6*
	MTART+I+G+F	*cox1*, *cytb*

MTART+I+G+F	*nad4L*, *nad1*, *nad4*, *nad5*

Table S3. The best partitioning schemes and models for Bayesian inference (BI) method based on four datasets selected by PartitionFinder.

Dataset	Models	Partitioning scheme
PCG12	GTR+I+G	*atp6*, *nad3*
	GTR+I+G	*atp8*, *nad6*, *nad2*
	GTR+I+G	*cox1*, *cytb*
	GTR+I+G	*cox3, cox2*
	GTR+I+G	*nad1*
	GTR+I+G	*nad4L*, *nad4*, *nad5*
PCG12R	GTR+I+G	*atp6*, *nad3*
	GTR+I+G	*atp8*, *nad6*, *nad2*
	GTR+I+G	*cox1*, *cytb*
	GTR+I+G	*cox3*, *cox2*
	GTR+I+G	*nad1*
	GTR+I+G	*nad4L*, *nad4*, *nad5*
	GTR+I+G	*rrnL*, *rrnS*
PCG123	GTR+I+G	*atp6*_codon1, *cox3*_codon1, *cox2*_codon1
	GTR+I+G	*atp6*_codon2, *nad3*_codon2, *nad6*_codon2, *nad2*_codon2, *atp8*_codon2
	GTR+I+G	*atp6*_codon3, *cytb*_codon3, *nad3*_codon3, *cox1*_codon3, *cox2*_codon3, *cox3*_codon3
	GTR+I+G	*nad3*_codon1, *nad6*_codon1, *atp8*_codon1, *nad2*_codon1
	HKY+I+G	*atp8*_codon3, *nad6*_codon3
	GTR+I+G	*cytb*_codon1, *cox1*_codon1
	GTR+I+G	*cox3*_codon2, *cox2*_codon2, *cox1*_codon2, *cytb*_codon2
	GTR+I+G	*nad1*_codon1
	GTR+I+G	*nad4*_codon2, *nad5*_codon2, *nad1*_codon2, *nad4L*_codon2
	HKY+I+G	*nad1*_codon3, *nad4*_codon3, *nad4L*_codon3, *nad5*_codon3
	GTR+G	*nad2*_codon3
	GTR+I+G	*nad5*_codon1, *nad4*_codon1, *nad4L*_codon1
PCG123R	GTR+I+G	*atp6*_codon1, *cox2*_codon1, *cox3*_codon1
	GTR+I+G	*cytb*_codon2, *cox1*_codon2, *cox3*_codon2, *cox2*_codon2, *nad3*_codon2, *atp6*_codon2
	GTR+I+G	*cytb*_codon3, *atp6*_codon3, *nad3*_codon3, *cox2*_codon3, *cox1*_codon3, *cox3*_codon3
	GTR+I+G	*nad3*_codon1, *nad6*_codon1, *nad2*_codon1, *atp8*_codon1
	GTR+I+G	*atp8*_codon2, *nad2*_codon2, *nad6*_codon2
	HKY+I+G	*atp8*_codon3, *nad6*_codon3
	GTR+I+G	*cox1*_codon1, *cytb*_codon1
	GTR+I+G	*nad1*_codon1
	GTR+I+G	*nad4*_codon2, *nad5*_codon2, *nad1*_codon2, *nad4L*_codon2
	HKY+I+G	*nad4*_codon3, *nad1*_codon3, *nad4L*_codon3, *nad5*_codon3
	GTR+G	*nad2*_codon3
	GTR+I+G	*nad5*_codon1, *nad4*_codon1, *nad4L*_codon1
	GTR+I+G	*rrnS*, *rrnL*
AA	MTREV+I+G	*atp6*, *cytb*
	MTREV+I+G	*nad5*, *atp8*, *nad6*, *nad4L*, *nad4*
	MTREV+I+G	*cox1*

MTMAM+I+G	*nad3*, *cox2*
MTREV+I+G	*cox3*, *nad1*
MTREV+I+G	*nad2*

Table S4. Nucleotide composition of the mitogenomes of 19 newly sequenced species.

Species	**Regions**	**Size(bp)**	**T(U)**	**C**	**A**	**G**	**AT(%)**	**GC(%)**	**AT skew**	**GC skew**
A1.	Full genome	16207	32.7	12.2	44.9	10.2	77.6	22.4	0.157	-0.088
	PCGs	10950	42.4	11.8	34.0	11.9	76.4	23.7	-0.109	0.003
	1st codon position	3650	35.6	11.0	36.8	16.7	72.4	27.7	0.017	0.206
	2nd codon position	3650	48.1	16.8	21.6	13.5	69.7	30.3	-0.380	-0.111
	3rd codon position	3650	43.4	7.5	43.6	5.4	87.0	12.9	0.003	-0.165
	tRNAs	1437	37.8	8.8	40.8	12.6	78.6	21.4	0.039	0.179
	rRNAs	1897	47.7	6.6	35.7	10.0	83.4	16.6	-0.143	0.204
	A+T rich-region	1919	26.0	11.5	51.3	11.3	77.3	22.8	0.328	-0.009
A2.	Full genome	15109	32.3	13.8	43.7	10.2	76.0	24.0	0.150	-0.147
	PCGs	10926	41.6	13.2	32.5	12.7	74.1	25.9	-0.123	-0.021
	1st codon position	3642	35.2	11.9	35.8	17.1	71.0	29.0	0.008	0.179
	2nd codon position	3642	47.9	17.7	20.8	13.6	68.7	31.3	-0.395	-0.131
	3rd codon position	3642	41.8	10.0	41.0	7.2	82.8	17.2	-0.010	-0.158
	tRNAs	1446	37.6	8.9	40.7	12.9	78.3	21.8	0.039	0.185
	rRNAs	1898	47.3	6.5	34.7	11.5	82.0	18.0	-0.154	0.279
	A+T rich-region	802	38.8	9.6	43.4	8.2	82.2	17.8	0.056	-0.077
E1.	Full genome	16987	34.7	11.8	42.8	10.7	77.5	22.5	0.106	-0.053
	PCGs	10902	42.8	11.7	33.3	12.3	76.1	24.0	-0.125	0.023
	1st codon position	3634	35.9	11.1	35.9	17.2	71.8	28.3	0.001	0.216
	2nd codon position	3634	48.4	17.2	20.9	13.6	69.3	30.8	-0.397	-0.116
	3rd codon position	3634	44.1	6.9	43.0	6.0	87.1	12.9	-0.012	-0.070
	tRNAs	1437	38.4	8.8	40.2	12.6	78.6	21.4	0.022	0.175
	rRNAs	1893	47.3	6.8	35.2	10.8	82.5	17.6	-0.147	0.229
	A+T rich-region	2715	38.2	9.0	40.8	12.0	79.0	21.0	0.033	0.142
E2.	Full genome	14749	33.1	12.7	44.5	9.7	77.6	22.4	0.147	-0.134
	PCGs	10932	43.1	11.8	33.3	11.8	76.4	23.6	-0.127	0.002
	1st codon position	3644	37.0	10.8	36.1	16.0	73.1	26.8	-0.013	0.193
	2nd codon position	3644	49.0	17.0	20.5	13.5	69.5	30.5	-0.411	-0.114
	3rd codon position	3644	43.1	7.5	43.5	5.9	86.6	13.4	0.004	-0.119
	tRNAs	1445	38.2	9.1	40.7	12.0	78.9	21.1	0.032	0.134
	rRNAs	2014	48.2	6.9	34.4	10.5	82.6	17.4	-0.168	0.208
	A+T rich-region	343	37.6	9.0	43.7	9.6	81.3	18.6	0.075	0.031
F.	Full genome	15563	33.1	12.2	44.8	10.0	77.9	22.2	0.150	-0.097
	PCGs	10947	42.8	11.9	32.8	12.5	75.6	24.4	-0.132	0.023
	1st codon position	3649	36.7	11.0	35.4	17.0	72.1	28.0	-0.017	0.215
	2nd codon position	3649	48.1	17.2	20.9	13.8	69.0	31.0	-0.393	-0.112
	3rd codon position	3649	43.8	7.5	42.1	6.6	85.9	14.1	-0.019	-0.060
	tRNAs	1429	38.0	9.4	40.4	12.2	78.4	21.6	0.030	0.133
	rRNAs	1942	46.9	6.7	35.1	11.3	82.0	18.0	-0.144	0.255
	A+T rich-region	1244	34.5	4.7	55.3	5.5	89.8	10.2	0.232	0.087
K.	Full genome	16321	32.2	13.6	43.6	10.6	75.8	24.2	0.151	-0.122
	PCGs	10899	42.2	11.8	34.5	11.6	76.7	23.4	-0.100	-0.009
	1st codon position	3633	35.8	11.1	36.5	16.6	72.3	27.7	0.009	0.196
	2nd codon position	3633	47.6	17.9	20.8	13.8	68.4	31.7	-0.392	-0.129
	3rd codon position	3633	43.1	6.3	46.3	4.3	89.4	10.6	0.036	-0.187
	tRNAs	1433	38.6	9.4	39.4	12.7	78.0	22.1	0.010	0.152
	rRNAs	1876	49.3	6.7	33.1	10.9	82.4	17.6	-0.197	0.242

	A+T rich-region	2083	32.8	17.2	30.9	19.1	63.7	36.3	-0.030	0.052
L1.	Full genome	17220	34.8	13.1	42.0	10.0	76.8	23.1	0.094	-0.135
	PCGs	10923	41.7	13.0	33.0	12.3	74.7	25.3	-0.116	-0.025
	1st codon position	3641	35.3	11.8	35.7	17.1	71.0	28.9	0.005	0.181
	2nd codon position	3641	48.6	17.2	20.8	13.4	69.4	30.6	-0.401	-0.126
	3rd codon position	3641	41.1	9.8	42.6	6.5	83.7	16.3	0.017	-0.201
	tRNAs	1420	38.8	8.3	39.9	13.0	78.7	21.3	0.013	0.221
	rRNAs	1895	46.1	7.4	34.8	11.8	80.9	19.2	-0.140	0.229
	A+T rich-region	2922	43.2	9.4	38.0	9.4	81.2	18.8	-0.064	0.000
L2.	Full genome	16840	35.8	11.6	42.4	10.2	78.2	21.8	0.084	-0.063
	PCGs	10932	43.3	11.5	33.4	11.7	76.7	23.2	-0.129	0.006
	1st codon position	3644	36.4	10.8	36.4	16.4	72.8	27.2	0.000	0.208
	2nd codon position	3644	48.3	17.9	20.7	13.2	69.0	31.1	-0.401	-0.151
	3rd codon position	3644	45.3	6.0	43.3	5.4	88.6	11.4	-0.023	-0.048
	tRNAs	1428	40.1	7.8	40.3	11.9	80.4	19.7	0.003	0.210
	rRNAs	1903	46.3	6.4	37.4	9.8	83.7	16.2	-0.107	0.210
	A+T rich-region	2541	41.5	8.9	37.4	12.2	78.9	21.1	-0.052	0.153
L3.	Full genome	16080	36.2	11.3	43.1	9.4	79.3	20.7	0.086	-0.091
	PCGs	10932	43.3	11.5	33.5	11.7	76.8	23.2	-0.129	0.006
	1st codon position	3644	36.4	10.8	36.4	16.4	72.8	27.2	0.000	0.208
	2nd codon position	3644	48.3	17.9	20.7	13.2	69.0	31.1	-0.401	-0.151
	3rd codon position	3644	45.4	6.0	43.3	5.4	88.7	11.4	-0.023	-0.051
	tRNAs	1428	40.1	7.8	40.3	11.9	80.4	19.7	0.003	0.210
	rRNAs	1903	46.3	6.5	37.4	9.8	83.7	16.3	-0.107	0.206
	A+T rich-region	1781	47.6	5.4	41.2	5.8	88.8	11.2	-0.072	0.035
L4.	Full genome	16040	33.6	12.9	44.1	9.3	77.7	22.2	0.135	-0.160
	PCGs	10926	42.4	12.4	33.2	12.0	75.6	24.4	-0.122	-0.015
	1st codon position	3642	36.1	11.2	36.3	16.4	72.4	27.6	0.003	0.188
	2nd codon position	3642	48.6	17.6	20.5	13.4	69.1	31.0	-0.408	-0.136
	3rd codon position	3642	42.6	8.4	42.8	6.3	85.4	14.7	0.003	-0.144
	tRNAs	1429	40.2	8.3	39.6	12.0	79.8	20.3	-0.007	0.183
	rRNAs	1901	47.1	6.6	35.1	11.3	82.2	17.9	-0.146	0.263
	A+T rich-region	1761	35.2	10.5	49.4	4.9	84.6	15.4	0.169	-0.360
S1.	Full genome	15970	34.6	12.0	43.6	9.8	78.2	21.8	0.115	-0.100
	PCGs	10908	42.3	11.4	34.5	11.8	76.8	23.2	-0.101	0.017
	1st codon position	3636	35.7	10.3	37.8	16.2	73.5	26.5	0.029	0.221
	2nd codon position	3636	48.3	16.5	21.9	13.3	70.2	29.8	-0.376	-0.106
	3rd codon position	3636	42.7	7.5	43.8	6.0	86.5	13.5	0.012	-0.112
	tRNAs	1439	39.1	8.1	40.7	12.2	79.8	20.3	0.020	0.199
	rRNAs	1978	47.0	6.4	35.9	10.7	82.9	17.1	-0.133	0.252
	A+T rich-region	1622	40.0	9.0	40.3	10.7	80.3	19.7	0.005	0.087
S2.	Full genome	16112	31.5	12.7	45.1	10.7	76.6	23.4	0.178	-0.084
	PCGs	10902	41.3	12.8	33.4	12.5	74.7	25.3	-0.106	-0.011
	1st codon position	3634	35.4	11.6	36.3	16.7	71.7	28.3	0.013	0.181
	2nd codon position	3634	47.4	17.7	21.2	13.7	68.6	31.4	-0.381	-0.128
	3rd codon position	3634	41.2	9.0	42.7	7.0	83.9	16.0	0.018	-0.122
	tRNAs	1425	38.0	9.3	40.6	12.1	78.6	21.4	0.034	0.128
	rRNAs	1968	48.3	6.9	34.4	10.4	82.7	17.3	-0.168	0.200
	A+T rich-region	1767	26.9	9.4	51.8	11.8	78.7	21.2	0.316	0.115
T1.	Full genome	16728	33.1	13.1	43.6	10.2	76.7	23.3	0.138	-0.126
	PCGs	10884	41.5	12.7	33.9	11.8	75.4	24.5	-0.101	-0.036
	1st codon position	3628	35.4	11.8	36.2	16.6	71.6	28.4	0.012	0.168
	2nd codon position	3628	47.4	17.9	21.1	13.6	68.5	31.5	-0.385	-0.136

	3rd codon position	3628	41.8	8.5	44.4	5.3	86.2	13.8	0.031	-0.226
	tRNAs	1435	37.6	9.4	40.3	12.8	77.9	22.2	0.035	0.151
	rRNAs	1895	48.7	6.6	33.6	11.1	82.3	17.7	-0.183	0.25
	A+T rich-region	2454	39	10.3	37.8	12.9	76.8	23.2	-0.016	0.113
	Full genome	16802	31.1	14.0	44.4	10.5	75.5	24.5	0.176	-0.146
	PCGs	10905	40.8	13.4	33.5	12.3	74.3	25.7	-0.098	-0.044
	1st codon position	3635	34.4	11.9	37.1	16.6	71.5	28.5	0.037	0.162
T2.	2nd codon position	3635	47.6	17.6	21.2	13.6	68.8	31.2	-0.384	-0.130
	3rd codon position	3635	40.4	10.6	42.4	6.6	82.8	17.2	0.024	-0.231
	tRNAs	1428	38.9	9.0	39.5	12.6	78.4	21.6	0.007	0.169
	rRNAs	1894	48.7	6.5	33.1	11.7	81.8	18.2	-0.191	0.283
	A+T rich-region	2501	29.1	13.4	44.7	12.9	73.8	26.3	0.211	-0.018
	Full genome	17151	30.9	13.8	44.4	10.8	75.3	24.6	0.179	-0.123
	PCGs	10956	41.4	12.9	32.8	12.8	74.2	25.7	-0.115	-0.003
	1st codon position	3652	36.3	11.4	35.3	17.0	71.6	28.4	-0.014	0.196
W.	2nd codon position	3652	47.3	17.5	21.1	14.0	68.4	31.5	-0.384	-0.110
	3rd codon position	3652	40.6	9.8	42.2	7.4	82.8	17.2	0.019	-0.137
	tRNAs	1438	37.6	9.3	39.9	13.1	77.5	22.4	0.030	0.170
	rRNAs	1886	47.7	7.3	33.8	11.3	81.5	18.6	-0.171	0.217
	A+T rich-region	2874	29.3	13.4	45.2	12.0	74.5	25.4	0.213	-0.056
	Full genome	16051	31.7	12.8	45.9	9.6	77.6	22.4	0.184	-0.146
	PCGs	10962	42.2	12.0	34.0	11.8	76.2	23.8	-0.108	-0.010
	1st codon position	3654	35.7	10.9	36.2	17.1	71.9	28.0	0.007	0.221
Y1.	2nd codon position	3654	47.9	18.0	20.6	13.5	68.5	31.5	-0.398	-0.142
	3rd codon position	3654	43.0	7.2	45.1	4.7	88.1	11.9	0.023	-0.207
	tRNAs	1441	37.5	9.3	39.4	13.7	76.9	23.0	0.024	0.193
	rRNAs	1880	48.3	6.8	34.4	10.5	82.7	17.3	-0.168	0.218
	A+T rich-region	1752	24.3	11.9	56.8	7.0	81.1	18.9	0.402	-0.263
	Full genome	16089	31.9	13.2	45.6	9.3	77.5	22.5	0.177	-0.174
	PCGs	10944	41.4	12.5	33.9	12.1	75.3	24.6	-0.099	-0.017
	1st codon position	3648	35.3	11.5	36.8	16.4	72.1	27.9	0.021	0.177
Y2.	2nd codon position	3648	47.8	17.5	20.8	13.8	68.6	31.3	-0.393	-0.117
	3rd codon position	3648	41.1	8.6	44.2	6.1	85.3	14.7	0.036	-0.169
	tRNAs	1435	38.2	9.3	40.6	11.9	78.8	21.2	0.031	0.125
	rRNAs	1880	48.9	6.3	33.7	11.1	82.6	17.4	-0.185	0.278
	A+T rich-region	1812	35.6	7.0	49.3	8.1	84.9	15.1	0.162	0.077
	Full genome	16720	33.4	12.7	43.6	10.3	77.0	23.0	0.133	-0.108
	PCGs	10908	42.2	12.2	33.7	11.9	75.9	24.1	-0.111	-0.010
	1st codon position	3636	35.8	10.8	36.7	16.7	72.5	27.5	0.012	0.214
Z1.	2nd codon position	3636	48.3	17.2	20.6	13.8	68.9	31.0	-0.402	-0.108
	3rd codon position	3636	42.4	8.6	43.8	5.3	86.2	13.9	0.017	-0.237
	tRNAs	1450	37.9	9.5	39.9	12.7	77.8	22.2	0.025	0.143
	rRNAs	1712	45.0	7.5	36.5	11.0	81.5	18.5	-0.105	0.190
	A+T rich-region	2607	33.8	11.1	44.2	11.0	78.0	22.1	0.133	-0.003
	Full genome	15166	33.2	13.1	43.8	9.9	77.0	23.0	0.138	-0.138
	PCGs	10902	42.3	12.2	33.5	12.0	75.8	24.2	-0.117	-0.009
	1st codon position	3634	35.7	11.1	36.3	17.0	72.0	28.1	0.009	0.209
Z2.	2nd codon position	3634	48.4	17.4	20.7	13.4	69.1	30.8	-0.400	-0.129
	3rd codon position	3634	42.8	8.2	43.3	5.6	86.1	13.8	0.006	-0.183
	tRNAs	1455	39.8	8.9	38.9	12.4	78.7	21.3	-0.011	0.168
	rRNAs	1912	47.9	6.9	34.0	11.2	81.9	18.1	-0.169	0.241
	A+T rich-region	819	38.5	13.2	38.3	10.0	76.8	23.2	-0.002	-0.137

Agnesiella irma (*A1.*); *Aguriahana wutyshana* (*A2.*); *Eupteryx* sp. (*E1.*); *Eurhadina rubra* (*E2.*), *Farynala starica* (*F.*); *Kuohzygia albolinea* (*K.*);

Limassolla dispunctata (*L1.*); *Limassolla fasciata* (*L2.*); *Limassolla galewskii* (*L3.*); *Limassolla uncata* (*L4.*); *Sannella crucifera* (*S1.*); *Shamala annulata* (*S2.*); *Thailocyba longilobula* (*T1.*); *Typhlocyba bilaminata* (*T2.*); *Warodia biguttata* (*W.*); *Yangida basnetti* (*Y1.*); *Yangisunda ramosa* (*Y2.*); *Zorka maculata* (*Z1.*) and *Zyginella mandali* (*Z2.*).

Table S5. Start and stop codons of the mitochondrial genomes. Note: *Agnesiella irma* (*A1.*); *Aguriahana wutyshana* (*A2.*); *Eupteryx* sp. (*E1.*); *Eurhadina rubra* (*E2.*), *Farynala starica* (*F.*); *Kuohzygia albolinea* (*K.*); *Limassolla dispunctata* (*L1.*); *Limassolla fasciata* (*L2.*); *Limassolla galewskii* (*L3.*); *Limassolla uncata* (*L4.*); *Sannella crucifera* (*S1.*); *Shamala annulata* (*S2.*); *Thailocyba longilobula* (*T1.*); *Typhlocyba bilaminata* (*T2.*); *Warodia biguttata* (*W.*); *Yangida basnetti* (*Y1.*); *Yangisunda ramosa* (*Y2.*); *Zorka maculata* (*Z1.*) and *Zyginella mandali* (*Z2.*).

Species / Gene	*nad2*	*cox1*	*cox2*	*atp8*	*atp6*	*cox3*	*nad3*	*nad5*	*nad4*	*nad4L*	*nad6*	*cytb*	*nad1*
A1.	ATA/TAA	ATG/TAA	ATT/T	TTG/TAA	ATG/TAA	ATG/T	ATT/TAA	ATT/T	ATG/TAG	ATG/TAA	ATT/TAG	ATG/TAA	ATT/TAA
A2.	ATA/TAA	ATG/TAG	ATT/T	TTG/TAA	ATG/TAA	ATG/T	ATT/TAA	ATT/T	ATG/TAA	ATG/TAA	ATC/TAA	ATG/TAA	ATT/TAA
E1.	ATA/TAA	ATG/TAA	ATT/T	TTG/TAA	ATG/TAA	ATG/T	ATT/TAA	TTG/T	ATG/TAA	ATG/TAA	ATT/TAA	ATG/TAG	ATT/TAA
E2.	ATA/TAA	ATG/TAA	ATA/T	TTG/TAA	ATG/TAA	ATG/T	ATT/TAG	ATG/T	ATG/TAA	ATG/TAA	ATA/TAA	ATG/TAG	ATT/TAA
F.	ATA/TAA	ATG/T	ATG/T	TTG/TAA	ATG/TAA	ATG/T	ATT/TAA	ATT/T	ATG/TAA	ATG/TAA	ATA/TAA	ATG/TAA	ATT/TAA
K.	ATA/TAA	ATG/T	ATT/T	TTG/TAA	ATG/TAA	ATG/T	ATA/TAA	ATA/T	ATG/TAA	ATG/TAA	ATT/TAA	ATG/TAG	ATT/TAA
L1.	ATG/TAG	ATG/T	ATT/T	TTG/TAA	ATG/TAA	ATG/T	ATA/TAA	ATT/TAA	ATG/T	ATG/TAA	ATA/TAA	ATG/TAA	ATT/TAA
L2.	ATT/TAG	ATG/T	ATT/T	TTG/TAA	ATG/TAA	ATG/TAA	ATA/TAA	ATA/TAA	ATG/T	ATG/TAA	ATA/TAA	ATG/TAA	ATT/TAA
L3.	ATT/TAG	ATG/T	ATT/T	TTG/TAA	ATG/TAA	ATG/TAA	ATA/TAA	ATA/TAA	ATG/T	ATG/TAA	ATA/TAA	ATG/TAA	ATT/TAA
L4.	ATA/TAG	ATG/T	ATC/T	TTG/TAA	ATG/TAA	ATG/T	ATA/TAA	ATA/TAA	ATG/T	ATG/TAA	ATA/TAA	ATG/TAA	ATT/TAA
S1.	ATA/TAA	ATG/TAA	ATA/T	TTG/TAA	ATA/TAA	ATG/T	ATT/TAA	ATA/T	ATG/TAA	ATG/TAA	ATT/TAA	ATG/TAG	ATT/TAA
S2.	ATA/TAG	ATG/T	ATG/T	TTG/TAA	ATG/TAA	ATG/T	ATT/TAA	ATT/T	ATG/TAA	ATG/TAA	ATA/TAA	ATG/TAG	ATT/TAA
T1.	ATA/TAA	ATG/T	ATT/T	ATA/TAA	ATG/TAA	ATG/T	ATT/TAA	ATA/T	ATG/T	ATG/TAA	ATA/TAA	ATG/TAG	ATT/TAA
T2.	ATT/TAA	ATG/TAA	ATC/T	TTG/TAA	ATG/TAA	ATG/T	ATT/TAA	ATG/T	ATG/TAA	ATG/TAA	ATA/TAA	ATG/TAG	ATT/TAA
W.	ATA/TAA	ATG/T	ATG/T	TTG/TAA	ATG/TAG	ATG/T	ATT/TAA	ATT/T	ATG/TAA	ATG/TAA	ATA/TAA	ATG/TAA	ATT/TAA
Y1.	ATA/TAA	ATG/TAA	ATT/T	TTG/TAA	ATG/TAA	ATG/T	ATT/TAA	ATT/T	ATG/TAA	ATG/TAA	ATA/TAA	ATG/TAA	ATT/TAA
Y2.	ATA/TAA	ATG/TAA	ATA/T	TTG/TAA	ATG/TAA	ATG/T	ATT/TAA	ATT/T	ATG/TAA	ATG/TAA	ATA/T	ATG/TAG	ATT/TAA
Z1.	ATA/TAA	ATG/TAA	ATA/T	ATA/TAA	ATG/TAA	ATG/T	ATT/TAG	ATT/T	ATG/TAA	ATG/TAA	ATA/TAA	ATG/TAA	ATT/TAA
Z2.	ATA/TAA	ATG/TAA	ATT/TAG	TTG/TAA	ATA/TAA	ATG/TAA	ATT/TAA	ATT/T	ATG/T	ATG/TAA	ATT/TAA	ATG/TAG	ATA/TAA
